# Pharmacological inhibition of Ref-1 enhances the therapeutic sensitivity of papillary thyroid carcinoma to vemurafenib

**DOI:** 10.1038/s41419-022-04550-0

**Published:** 2022-02-08

**Authors:** Linfei Hu, Jun Zhang, Mengran Tian, Ning Kang, Guangwei Xu, Jingtai Zhi, Xianhui Ruan, Xiukun Hou, Wei Zhang, Jiaoyu Yi, Weike Ma, Luchen Chang, Tao Tang, Xiangqian Zheng, Xi Wei, Ming Gao

**Affiliations:** 1 Department of Thyroid and Neck Tumor, Tianjin Medical University Cancer Institute and Hospital, National Clinical Research Center for Cancer, Key Laboratory of Cancer Prevention and Therapy, Tianjin’s Clinical Research Center for Cancer, 300060 Tianjin, China; 2 Department of Breast Cancer, Tianjin Medical University Cancer Institute and Hospital, Key Laboratory of Breast Cancer Prevention and Therapy, Key Laboratory of Cancer Prevention and Therapy, Tianjin’s Clinical Research Center for Cancer, National Clinical Research Center of Cancer, Tianjin Medical University Cancer Institute and Hospital, 300060 Tianjin, China; 3 Department of Otolaryngology-Head and Neck Surgery, Tianjin First Center Hospital, Nankai District of Tianjin, Institute of Otolaryngology of Tianjin, Key Laboratory of Auditory Speech and Balance Medicine, Key Clinical Discipline of Tianjin (Otolaryngology), Otolaryngology Clinical Quality Control Centre, 300100 Tianjin, China; 4 Department of Diagnostic and Therapeutic Ultrasonography, Tianjin Medical University Cancer Institute and Hospital, National Clinical Research Center for Cancer, Key Laboratory of Cancer Prevention and Therapy, Tianjin’s Clinical Research Center for Cancer, 300060 Tianjin, China; 5 Department of Hepatobiliary Surgery, Affiliated Hospital of North Sichuan Medical College, 637000 Nanchong, China; 6 Department of Breast and Thyroid Surgery, Tianjin Union Medical Center, 300121 Tianjin, China

**Keywords:** Cancer therapeutic resistance, Head and neck cancer

## Abstract

The use of the BRAF inhibitor vemurafenib exhibits drug resistance in the treatment of thyroid cancer (TC), and finding more effective multitarget combination therapies may be an important solution. In the present study, we found strong correlations between Ref-1 high expression and BRAF mutation, lymph node metastasis, and TNM stage. The oxidative stress environment induced by structural activation of BRAF upregulates the expression of Ref-1, which caused intrinsic resistance of PTC to vemurafenib. Combination inhibition of the Ref-1 redox function and BRAF could enhance the antitumor effects of vemurafenib, which was achieved by blocking the action of Ref-1 on BRAF proteins. Furthermore, combination treatment could cause an overload of autophagic flux via excessive AMPK protein activation, causing cell senescence and cell death in vitro. And combined administration of Ref-1 and vemurafenib in vivo suppressed PTC cell growth and metastasis in a cell-based lung metastatic tumor model and xenogeneic subcutaneous tumor model. Collectively, our study provides evidence that Ref-1 upregulation via constitutive activation of BRAF in PTC contributes to intrinsic resistance to vemurafenib. Combined treatment with a Ref-1 redox inhibitor and a BRAF inhibitor could make PTC more sensitive to vemurafenib and enhance the antitumor effects of vemurafenib by further inhibiting the MAPK pathway and activating the excessive autophagy and related senescence process.

## Introduction

Papillary thyroid cancer (PTC) is the most common pathological subtype of thyroid cancer (TC), and its BRAF mutation rate, especially the point mutation of valine at the 600 positions to glutamate, i.e., BRAFV600E, can reach >80% in Asian populations, which creates a very favorable condition for targeted therapy in this population. Currently, the BRAF inhibitors (BRAFis) approved by the Food and Drug Administration mainly include sorafenib, regorafenib, vemurafenib, dabrafenib, and encorafenib [1]; the last three are mainly used to treat patients with advanced melanoma, which also harbors a high BRAF mutation rate, and among them, vemurafenib, a BRAF^V600E^-specific small-molecule inhibitor, has significantly changed therapeutic prospects and been shown to quickly inhibit the growth of melanoma and control malignant tumor progression in most patients [2, 3]. To date, the application of vemurafenib in advanced PTC is still in clinical trials, showing potential clinical application value [4–6]. Experiments in vitro have revealed poor vemurafenib treatment sensitivity and early resistance [7–9] in PTC cell lines. Under these circumstances, higher drug concentrations are required to achieve a satisfactory inhibitory effect, which tremendously increases the risk of drug toxicity to achieve the same expected effect in vivo. At present, multiple studies have reported the mechanism of drug resistance in tumors, including activity attenuation of immune cells [10], bypass of mitogen-activated protein kinase (MAPK) pathway activity [11], loss of negative feedback [12, 13], and increased antiapoptotic protein Bcl2 expression [14]. However, there is no final conclusion at present on what causes an inadequate therapeutic index in PTC, melanoma, or other cancers regarding the mechanism of resistance, and novel mechanisms and strategies for combination drugs still need to be exploited.

Redox factor-1 (Ref-1), also known as apurinic/apyrimidinic endonuclease 1 (APE1), is a highly conserved functional enzyme that has a redox function that regulates the activity of a variety of important transcription factors and has nucleic acid endonuclease activity, allowing Ref-1 to function as a DNA repair enzyme. In the past, numerous studies [15, 16] have verified that Ref-1 is involved in the drug resistance process as a DNA repair enzyme in various tumors, and an increasing number of reports [17–19] have shown that Ref-1 can also regulate drug resistance through its redox-dependent activity. In a proteomic analysis, Salzano et al. [20] identified 13 differentially expressed proteins between differentiated rat thyroid cell line and derived undifferentiated cell line nuclear extracts, and Ref-1, one of the proteins that might be involved in a transcriptional mechanism, was suspected to be associated with the occurrence of TC. Liu et al. [21] indicated that the expression of Ref-1 was decreased during the early stage of melanoma, while in later stages during the transformation into malignant metastatic melanoma with an increase in the expression level of redox activity of Ref-1. Another study revealed that upregulation of Ref-1 expression promoted melanoma resistance to BRAFis [22]. These previous reports raise the possibility that Ref-1 may be an important causative factor in the differential response to drug therapy between PTC and melanoma.

In the present study, we found that Ref-1 was highly expressed in TC and had a positive correlation with BRAF mutation and induced oxidation environment by MAPK pathway constitutive activation. A Ref-1 redox inhibitor could enhance the abilities of vemurafenib to inhibit proliferation and metastasis and promote apoptosis differentiation in vitro and in vivo. Additionally, the inhibitor could synergize with vemurafenib to cause cell senescence. Mechanistic studies of PTC cells in vitro demonstrated that Ref-1 reduced the antitumor effects of vemurafenib by binding to the BRAF protein and combined therapy with vemurafenib and E3330 caused cell senescence by overburdening autophagic flow.

## Results

### Clinical Ref-1 expression was related to an inferior prognosis and BRAF mutation in PTC

**Fig. 1:**
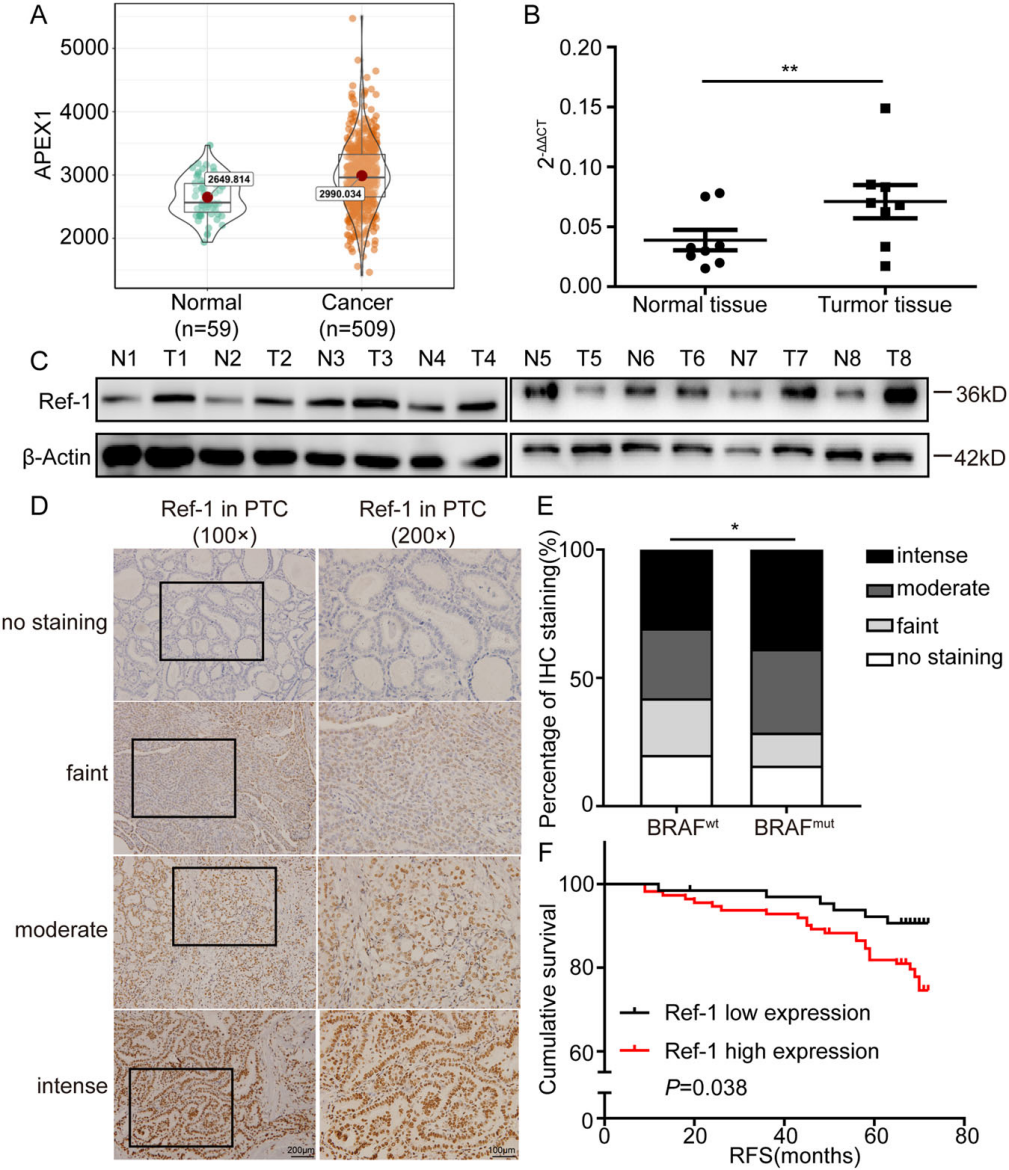
Ref-1 expression was upregulated in PTC patient samples. **A** Amplification alteration of the APEX1 gene in thyroid cancer and normal tissue samples from The Cancer Genome Atlas (TCGA) database. **B** Real-time PCR detection of the mRNA expression level of Ref-1 in tumor and normal tissue samples from 16 patients. **C** Western blot detection of the protein expression level of Ref-1 in tumor and normal tissue specimens from 8 patients. **D** Representative immunohistochemical staining for Ref-1 in PTC specimens. **E** Percentages of samples with different BRAF mutation statuses and specific Ref-1 expression levels among 178 PTC cases. **F** RFS analysis of groups based on the high and low expression of Ref-1 among 178 PTC cases. *P < 0.05, **P < 0.01.

To determine the expression of Ref-1 in PTC, we first extracted sample data of 568 PTC patients (509 PTC samples and 59 normal samples) from The Cancer Genome Atlas (TCGA) database and then conducted quantitative real-time PCR (qPCR) and western blot analyses of normal and tumor fresh tissue samples from 16 PTC patients and 8 PTC patients, respectively. The analyses revealed that the mRNA and protein levels of Ref-1 were both higher in tumor tissue than in normal tissue (Fig. 1A–C). To investigate the effects of Ref-1 expression levels on PTC patients, immunohistochemical (IHC) staining of 178 PTC samples, which were divided into four groups based on the Ref-1 staining score, was conducted (Fig. 1D). IHC scoring and clinicopathological data analysis showed that high expression of Ref-1 was positively correlated with BRAF mutation and lymph node metastasis (Table 1 and Fig. 1E) but not associated with patient sex, age, tumor diameter, number of lesions, T stage, or TNM stage (AJCC Eighth) and indicated that patients with negative Ref-1 staining had longer relapse-free survival than those with positive staining (Fig. 1F). PCR and western blot analysis of thyroid cell lines showed notably increased Ref-1 expression in BRAF^V600E^ cells (Supplemental Fig. 1A, B).

**Table 1:** Analysis of Ref-1 expression and clinicopathological features in PTC.

Vairables	Ref-1 expression			*P* value
	Negative/low	Moderate/high		
Age, years				
<55	43		68	0.428
≥55	22		45	
Gender				
Male	17		33	0.663
Female	48		80	
Multifocality				
No	46		79	0.904
Yes	19		34	
BRAF mutation				
No	40		45	0.005
Yes	25		68	
T stage				
T1 + T2	59		101	0.767
T3 + T4	6		12	
LNM				
No	38		48	0.040
Yes	27		65	
TNM stage				
I–II	62		103	0.296
III–IV	3		10	

### An intrinsic high level of Ref-1 contributed to depressed vemurafenib sensitivity in BRAF^V600E^ PTC

**Fig. 2:**
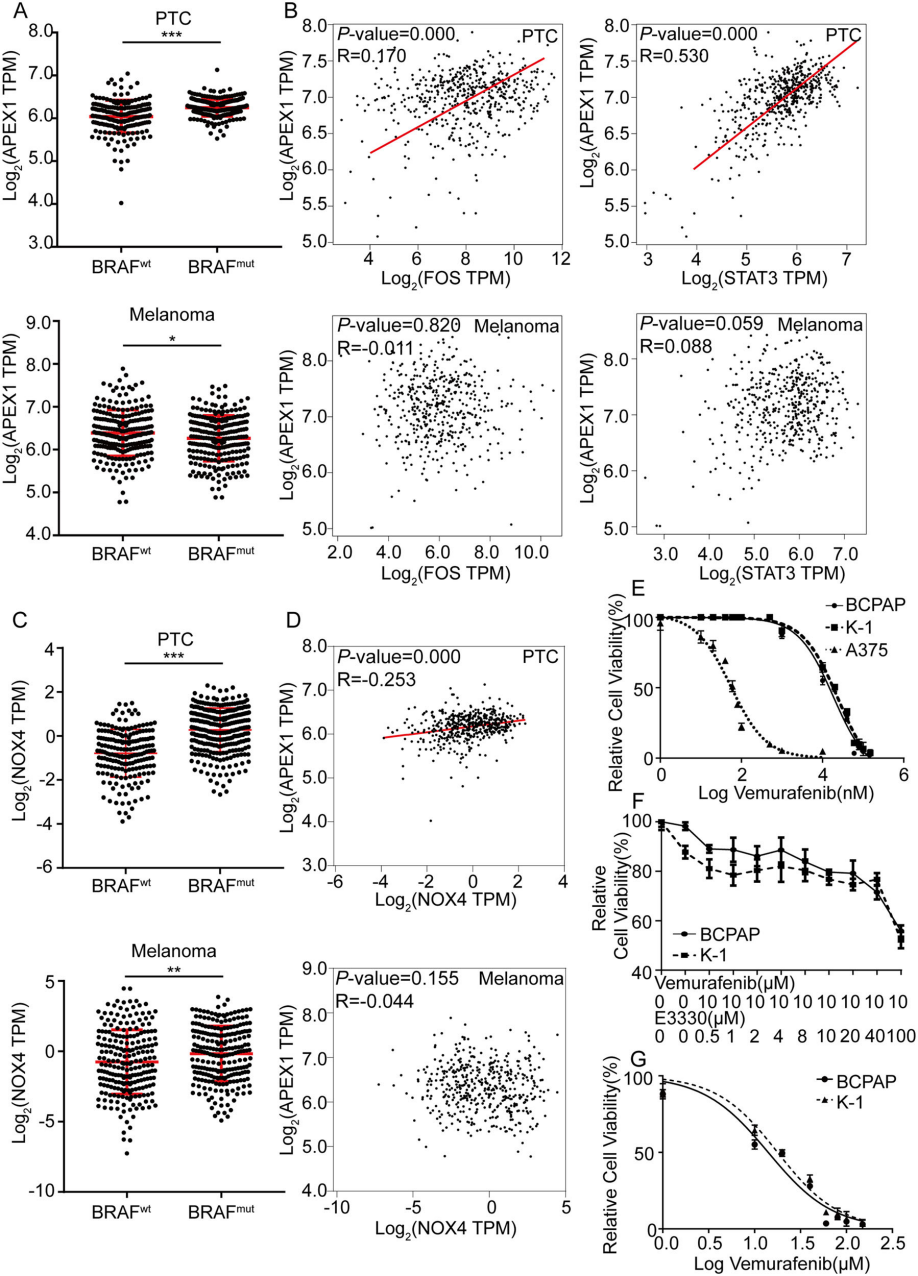
Resistance to vemurafenib in PTC was related to upregulated Ref-1 expression. **A** Analysis of data from the TCGA database for Ref-1 mRNA expression levels in BRAF^wt^ and BRAF^mut^ PTC (upper) and melanoma (bottom) cases. **B** Correlation analysis of Ref-1 and MAPK pathway targeting genes expression in PTC (upper) and melanoma (bottom) cases based on the TCGA database. **C** Analysis of data from the TCGA database for NOX4 mRNA expression levels in BRAF^wt^ and BRAF^mut^ PTC (upper) and melanoma (bottom) cases. **D** Correlation analysis of Ref-1 and NOX4 mRNA expression levels in PTC (upper) and melanoma (bottom) cases based on the TCGA database. **E** Vemurafenib IC_50_ detection by a CCK-8 assay in BCPAP, K-1, and A375 cells. **F** Cell proliferation ability detection by a CCK-8 assay after vemurafenib (10 μM) and E3330 (different concentration gradient) pretreatment in BCPAP and K-1 cells. **G** Vemurafenib IC_50_ detection after E3330 pretreatment by a CCK-8 assay in BCPAP and K-1 cells. *P < 0.05, **P < 0.01. ***P < 0.001.

Then, sample data from 487 PTC patients (198 with BRAF^wt^ and 289 with BRAF^mut^) and 469 melanoma patients (229 with BRAF^wt^ and 240 with BRAF^mut^) extracted from the TCGA were divided into two groups according to the BRAF mutation status. Gene expression analysis revealed that BRAF mutation elevated the mRNA level of Ref-1 in PTC (*P* < 0.0001), while melanoma exhibited the opposite pattern (*P* = 0.0115) (Fig. 2A). The observation of positive correlations between the expression of Ref-1 and markers of MAPK pathway activation in PTC but not in melanoma reconfirmed the patterns (Fig. 2B). BRAF mutation is closely related to intracellular oxidative stress, we analyzed the expression of ROS-producing enzyme NOX4 and its relationship with APEX1 in the TCGA database. The results showed that BRAF mutation was accompanied by high expression of NOX4, and the expression level of the latter is positively correlated with the expression of redox factor APEX1 (Fig. 2C, D). To further verify whether the increased expression of Ref-1 is involved in BRAFi resistance in BRAF^V600E^ PTC, we treated PTC cell lines (BCPAP and K-1, both harboring the BRAF^V600E^ mutation) and a melanoma cell line (A375, harboring the BRAF^V600E^ mutation) with different concentrations of vemurafenib for IC50 testing, and the results showed a higher sensitivity in the melanoma cell line (IC50_A375_ = 56.46 nM) than in the PTC cell lines (IC50_BCPAP_ = 31.36 μM, IC50_K-1_ = 36.62 μM) (Fig. 2E). Next, we used different concentrations of E3330 combined with 10 μM vemurafenib to treat TC cell lines BCPAP and K-1, and observed that vemurafenib can significantly increase the inhibitory effect of vemurafenib on cell proliferation at a concentration of 50 μM (Fig. 2F). Then we treated BCPAP and K-1 cells with the Ref-1 redox inhibitor E3330 for 24 h and then repeated the IC50 testing procedure for vemurafenib. To our surprise, pretreatment with E3330 distinctly increased the sensitivity of the PTC cell lines to vemurafenib (IC50_BCPAP_ = 14.06 μM, IC50_K-1_ = 17.80 μM) (Fig. 2G). Based on the above analysis, the expression level of Ref-1 was increased in BRAF^V600E^ PTC with active redox systems, which indicates that Ref-1 is involved in a mechanism for intrinsic resistance to a BRAFi in PTC.

### The Ref-1 redox inhibitor enhanced the sensitivity of BRAF^V600E^ PTC to vemurafenib in vitro

**Fig. 3:**
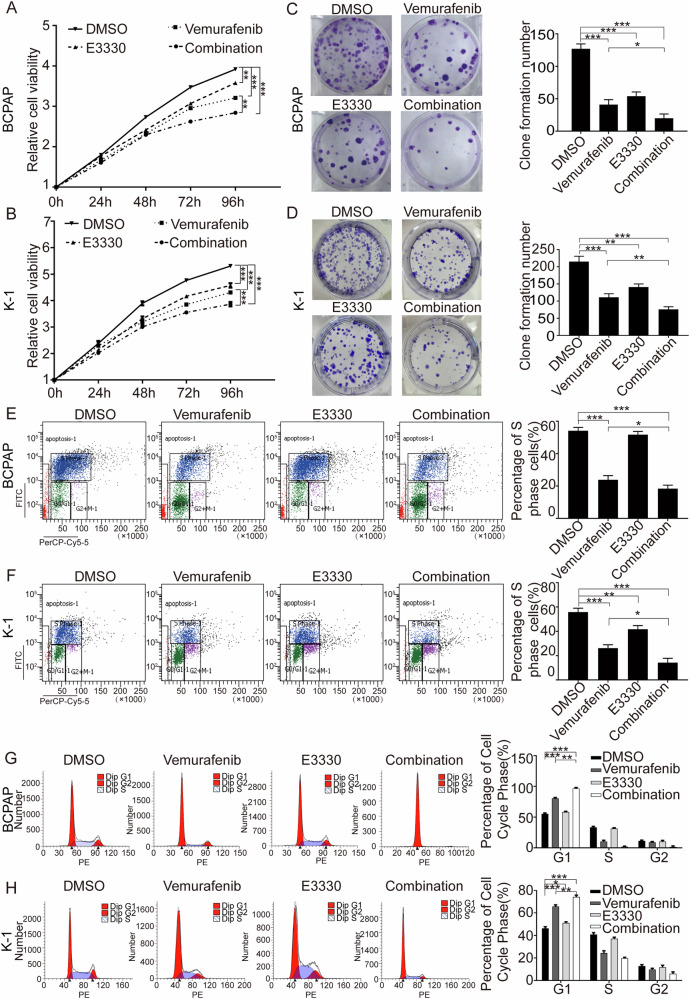
E3330 enhanced the antiproliferative capacity of vemurafenib in BCPAP and K-1 cells. **A**, **B** Cell viability detection by a CCK-8 assay was used to quantify cell proliferation in BCPAP and K-1 cell lines. **C**, **D** Cell proliferation detection by colony-formation assay in BCPAP and K-1 cell lines. **E**, **F** Cell division detection by BrdU assay in BCPAP and K-1 cell lines. **G**, **H** The cell cycle distribution detection by flow cytometric assay in BCPAP and K-1 cell lines. BCPAP and K-1 cell lines were pretreated with DMSO, vemurafenib (10 μM), E3330 (50 μM), and combination (10 μM vemurafenib + 50 μM E3330), respectively. *P < 0.05, **P < 0.01, ***P < 0.001.

**Fig. 4:**
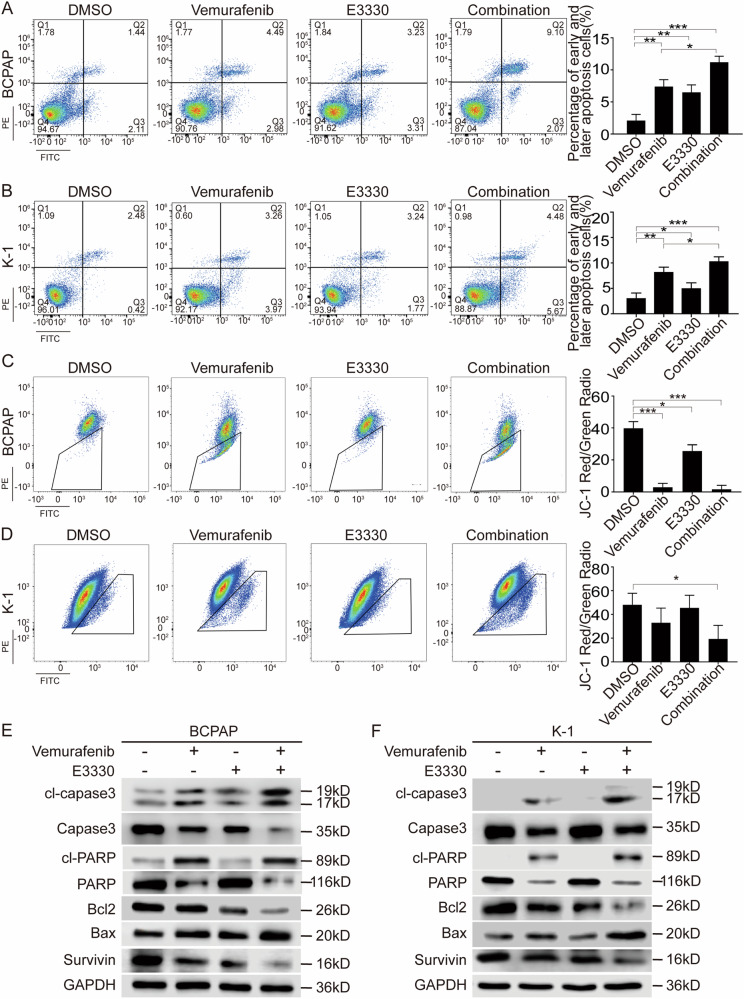
E3330 enhanced the proapoptotic capacity of vemurafenib in BCPAP and K-1 cells. **A**, **B** The cell apoptosis rate measured by flow cytometric analysis in BCPAP and K-1 cell lines. **C**, **D** The mitochondrial membrane potential evaluation by detecting JC-1 content in BCPAP and K-1 cell lines. **E**, **F** Mitochondrial apoptosis pathway-associated protein expression detection by western blot assay in BCPAP and K-1 cell lines. BCPAP and K-1 cell lines were pretreated with DMSO, vemurafenib (10 μM), E3330 (50 μM), and combination (10 μM vemurafenib + 50 μM E3330), respectively. *P < 0.05, **P < 0.01, ***P < 0.001.

To assess the effect of vemurafenib and further evaluate the effect of combination treatment on PTC in vitro, BCPAP and K-1 cells, which harbor BRAF^V600E^, were exposed to vemurafenib or/and E3330 to detect the effects of the two inhibitors. As expected, CCK-8 and clone formation experiments revealed that E3330 could enhance the antitumor effect of vemurafenib (Fig. 3A–D). Using flow cytometry to detect BrdU, we found that the proportion of S-phase cells in the combination group was significantly reduced, which indicated that E3330 could enhance the antiproliferative effect of vemurafenib, and the results of cell cycle experiments suggested that proliferation was mainly arrested in the G1 phase (Fig. 3E–H). Next, apoptosis evaluation showed that E3330 could significantly enhance the proapoptotic property of vemurafenib (Fig. 4A, B), and this effect was mainly achieved via the mitochondrial apoptosis pathway (Fig. 4C, D), as determined by assessing Bcl2, Bax, cleaved-PARP (cl-PARP), cleaved-caspase3 (cl-caspase3) and Survivin (Fig. 4E, F). Furthermore, transwell assay results showed that E3330 also enhanced the capacity of vemurafenib to inhibit migration and invasion (Supplemental Fig. 2A–D) and to affect E-cadherin and Vimentin levels in BCPAP and K-1 cell lines (Supplemental Fig. 2E, F). We analyzed the combined effects on angiogenesis and tumor differentiation in vivo and in vitro. The results show that both vemurafenib and E3330 could inhibit the angiogenesis of thyroid tumors and the combination treatment has a more significant inhibition effect in the angiogenesis phenotype (Supplemental Fig. 3A, B). In cell differentiation function analysis, both vemurafenib and E3330 can reduce the level of ROS in TC cells to a certain extent, and the combined treatment can further reduce the level of ROS, and the reduced ROS level can promote the differentiation of TC cells and induce cell differentiation (Supplemental Fig. 4A–C). The above results demonstrated that in combination therapy, E3330 played a role in sensitizing BRAF^V600E^ PTC cells to vemurafenib.

### Ref-1 regulated sensitivity to vemurafenib via a redox-dependent mechanism in PTC

**Fig. 5:**
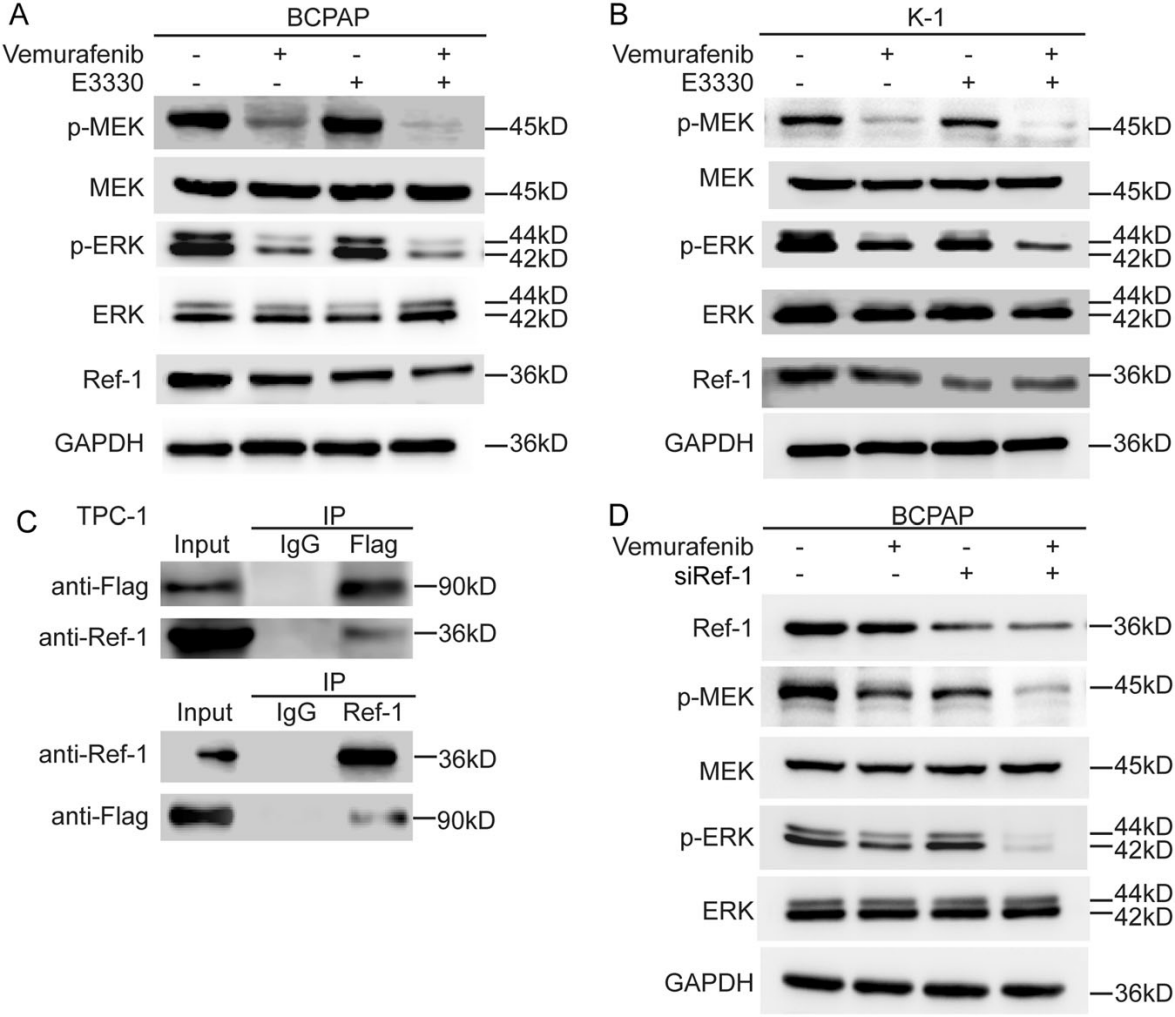
Ref-1 promoted the maintenance of mutant BRAF function. **A**, **B** Western blot detection of MAPK pathway activation in DMSO, vemurafenib (10 μM), E3330 (50 μM), and combination (10 μM vemurafenib + 50 μM E3330) groups. **C** The mode of action of Ref-1 and BRAF determination by a Co-IP assay using TPC-1 cells with a BRAF^V600E^ Tet-on system. **D** MAPK pathway activation detection by western blot assay after small interfering RNA targeting of Ref-1 with vemurafenib treatment.

To further investigate the sensitizing mechanism of E3330, we detected the levels of the BRAF downstream proteins p-MEK and p-ERK in the classical MAPK pathway. Western blot analysis showed that the levels of phosphorylated MEK and ERK were significantly lower than those seen with vemurafenib treatment alone (Fig. 5A, B), so we speculated that Ref-1 promotes or maintains the function of BRAF. Subsequently, we selected the BRAF wild-type cell line TPC-1 to construct a BRAF^V600E^-overexpressing stable cell line for Co-IP and the BCPAP cell line for small interfering RNA knockdown experiments to verify the mechanism linking BRAF and Ref-1. The results showed that Ref-1 played a role in maintaining BRAF function by binding with BRAF (Fig. 5C, D).

### Senescence was involved in the dual-suppression outcome of Ref-1 and BRAF through an overloaded autophagic flux effect

**Fig. 6:**
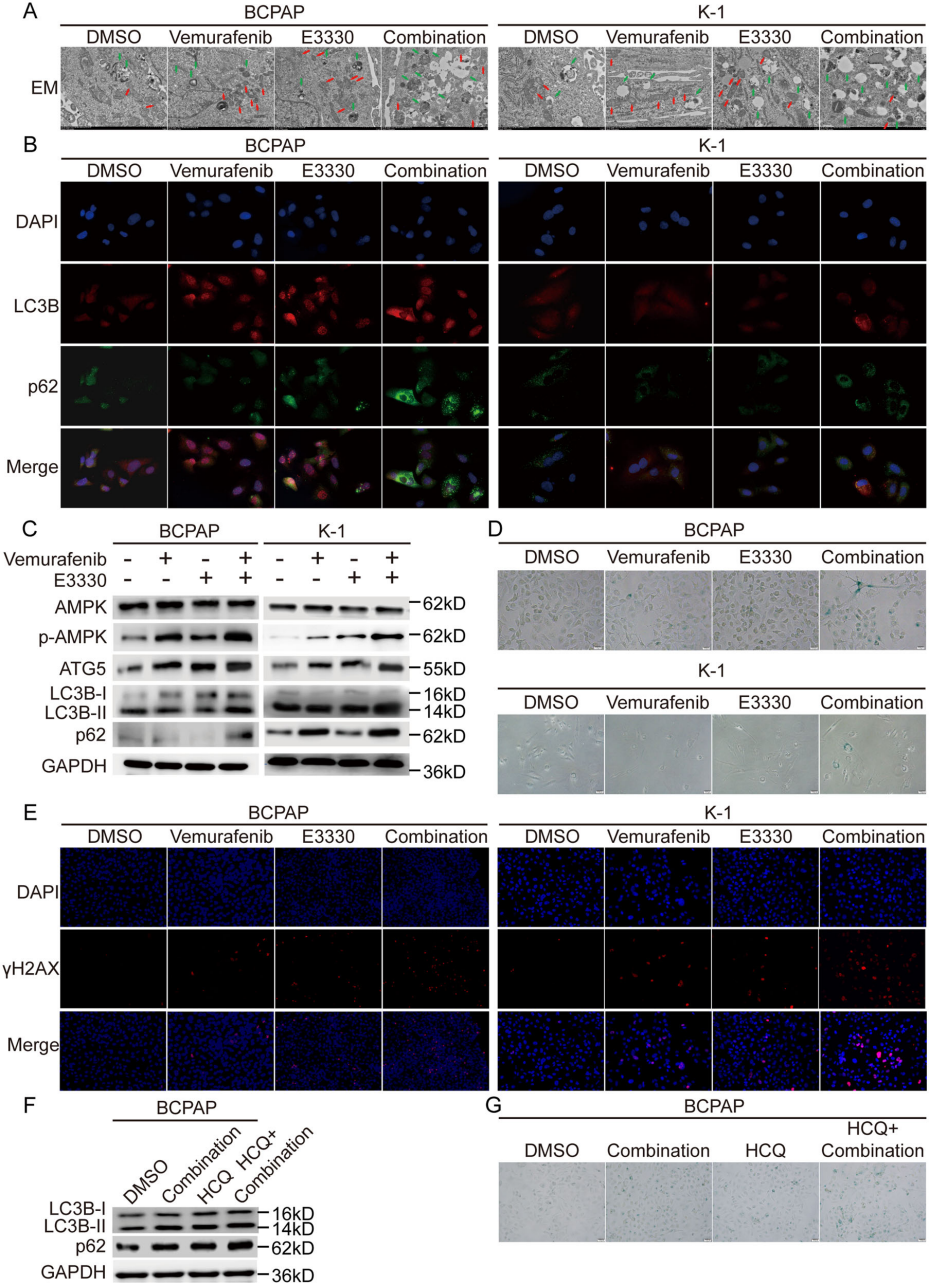
Combined Ref-1 redox inhibitor and vemurafenib treatment induced autophagic flow overload and caused senescence in BCPAP and K-1 cells. **A** Representative electron micrographs of BCPAP and K-1 cell lines. The green arrows indicate lysosome and the red arrows indicate autophagosome. **B** Immunofluorescence detection of the autophagic substrates LC3B and p62 in BCPAP and K-1 cell lines. **C** Western blot detection of the autophagic substrates LC3B and p62 in BCPAP and K-1 cell lines. **D** Cellular senescence detection by β-galactosidase staining in BCPAP and K-1 cell lines. **E** Immunofluorescence detection of γH2AX in BCPAP and K-1 cell lines. **F** Western blot detection of the autophagic substrates LC3B and p62 in BCPAP and K-1 cell lines. **G** Cellular senescence detection by β-galactosidase staining in BCPAP and K-1 cell lines. BCPAP and K-1 cell lines in **A**–**E** were pretreated with DMSO, vemurafenib (10 μM), E3330 (50 μM), and combination (10 μM vemurafenib + 50 μM E3330), respectively. BCPAP and K-1 cell lines in **F**, **G** were pretreated with DMSO, combination (10 μM vemurafenib + 50 μM E3330), HCQ, and combination + HCQ, respectively.

A number of studies [23–26] have shown that vemurafenib can promote the occurrence of protective autophagy while inducing limited apoptosis, which is an important reason for treatment resistance. In fact, the duration and intensity of the autophagosome signal can cause completely different outcomes that determine whether tumors survive or die [27]. To verify the autophagic phenomenon induced by vemurafenib treatment and identify the final trend in autophagy, we observed the formation and fusion of autophagosomes, lysosomes, and the distribution and expression level of the common autophagy marker in the meanwhile. In the vemurafenib group and E3330 group, autophagosomes, lysosomes, and other organelles fused into autophagolysosomes at different stages of formation. And in the combination group, we found that following the increase of phagocytosis and fusion, more and larger vacuoles were formed in the cells, occupying most of the cytoplasmic space, and there were few organelles left in the cells (Fig. 6A). In addition, enhanced autophagy induced by vemurafenib was notably present, and combined treatment with vemurafenib and the Ref-1 inhibitor strikingly induced LC3B, p62 colocalization and accumulation, AMP-activated protein kinase (AMPK) activation, ATG5 expression increasing in the BCPAP and K-1 cell lines, which indicated an incomplete or excessive autophagy process (Fig. 6B, C). Research has reported that basal autophagy is essential to maintain stem cell quiescence by preventing aging, while the accumulation of autophagic substrates can cause cell senescence and death [28, 29]. Subsequently, we performed senescence staining and γH2AX marking in BCPAP and K-1 cell liens after single or combined treatment with the two inhibitors. A highly significant senescence phenotype that occurred in the combined treatment strategy was observed (Fig. 6D, E). Then, HCQ (hydroxychloroquine), a lysosomal autophagy inhibitor that can block autophagy substrate conversion, was used to further validate the senescence phenotype caused by the impaired autophagy process. (Fig. 6F, G).

### The Ref-1 redox inhibitor enhances the sensitivity of PTC cells to vemurafenib treatment in vivo

**Fig. 7:**
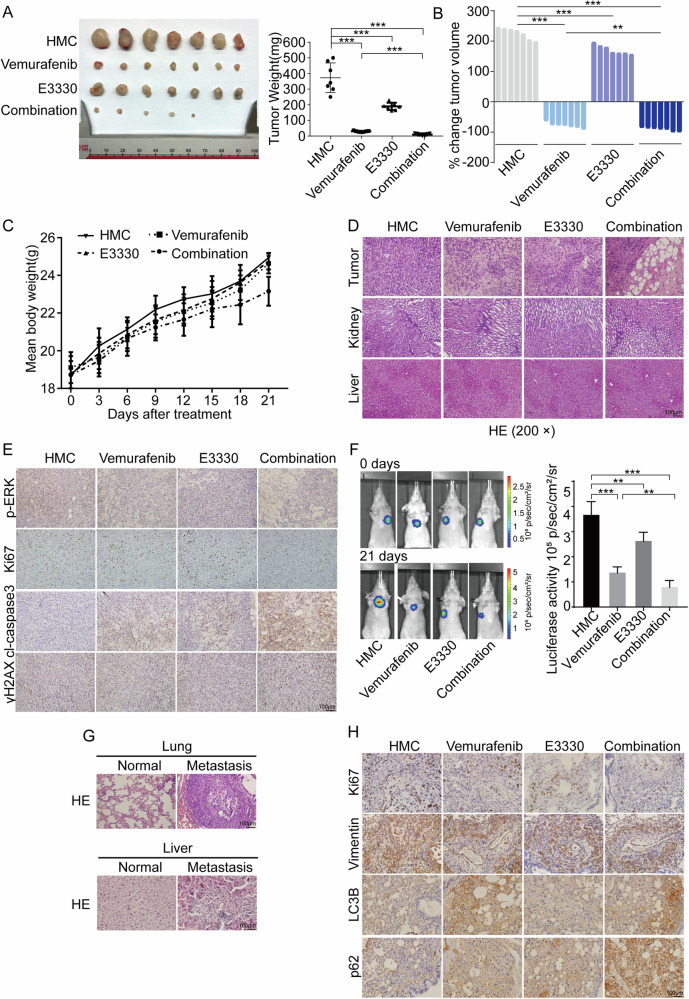
Vemurafenib combined with E3330 treatment offered a relatively promising therapeutic strategy in vivo. **A** Representative images of dissected mouse subcutaneous tumors after HMC, vemurafenib, E3330, or combination (vemurafenib + E3330) treatment for 21 days. **B** The weights of mouse subcutaneous tumors after different treatments for 21 days. **C** Change in tumor volume after HMC, vemurafenib, E3330, or combination (vemurafenib + E3330) treatment for 21 days. **D** The body weight changes of mice measured every 3 days after different treatments. **E** Representative H&E staining of tumors, livers, and kidneys after different treatments. **F** Representative immunohistochemical staining for p-ERK, Ki-67, cl-caspase3, and γH2AX after different treatments. **G** Representative images of mouse metastatic tumors after HMC, vemurafenib, E3330, or combination (vemurafenib + E3330) treatment for 21 days. **H** Representative H&E staining of metastatic tumors in the lungs and livers. **I** Representative immunohistochemical staining for Ki-67, Vimentin, LC3B, and p62 after different treatments. **P < 0.01, ***P < 0.001.

Xenogeneic subcutaneous tumor model experiments showed that combined vemurafenib and Ref-1 inhibitor treatment significantly inhibited the growth and progression of xenogeneic subcutaneous tumors (Fig. 7A). Moreover, the combination group showed the effect of complete reduction for PTC, and the combination strategy is expected to enable complete remission of early-stage tumors (Fig. 7B). Increased rate of body weight and hematoxylin–eosin (H&E) staining of liver and kidney tissue samples showed a reliable safety coefficient (Fig. 7C, D). IHC staining of tumor tissue samples showed decreased Ki-67 and p-ERK expression and increased cl-caspase3 and γH2AX expression (Fig. 7E).

In lung metastasis animal experiment analysis, compared with single-drug treatment, combined vemurafenib and Ref-1 inhibitor treatment significantly inhibited the growth of metastatic tumors and tumor progression (Fig. 7F). Additionally, in addition to lung metastasis, a liver metastasis case occurred in both the control group and the vemurafenib group during drug administration, indicating that E3330 might have a superior inhibitory effect on tumor metastasis (Fig. 7G). IHC staining of lung metastatic tumor tissue sections indicated decreased cell proliferation number as determined by Ki-67 staining; depressed cell metastasis ability, as determined by Vimentin staining; and overloaded autophagy flux, as determined by LC3B and p62 staining (Fig. 7H). These results were consistent with the results of the in vitro cell experiments, indicating that E3330 could exert sensitization and synergistic effects in combination with vemurafenib on tumor progression in vivo.

## Discussion

In addition to the high recurrence rate and reduced survival rate, the dedifferentiation of thyroid cells and decline in functional gene expression are also closely related to BRAF mutation [30]. BRAF^V600E^, which accounts for 90% of BRAF mutations in human cancers [31] and has high catalytic activity alone, does not require dimerization for function [32, 33], which is one of the important reasons for the high degree of malignancy of BRAF-mutant PTC. As a competitive inhibitor, vemurafenib selectively binds to the ATP (adenosine triphosphate)-binding site of the kinase BRAF, thereby reducing ATP entry to block MAPK pathway activation [31].

In recent years, targeted therapies for PTC have changed focus from inhibiting tumor neovascularization to targeting specific gene mutations. In a preliminary analysis, we observed lower sensitivity to vemurafenib in PTC patients and cell lines than in melanoma patients and a melanoma cell line. At present, according to published reports, there are two main reasons for PTC patients developing resistance to vemurafenib therapy: one is the compensatory or bypass activation of MAPK pathway-related proteins, and the other is the heterogeneity of cells with various genetic backgrounds in the same tumor tissue and different types of tumor cells [34]. The former mechanism is mainly classified as acquired resistance and has been revealed to involve multifarious signaling pathways and occur in various tumors. The latter is generally considered intrinsic resistance, but the mechanism remains unclear.

In fact, the thyroid is an organ that needs to synthesize oxidative thyroid hormones, and this process inevitably generates a large amount of reactive oxygen free radicals. A number of studies have shown that TC tissues have higher levels of oxidants than that of normal tissues [35, 36]. The production of ROS in TC mainly derives from NADPH oxidase and mitochondria, in which NOX4 is the only NADPH oxidase with constitutive ROS generation activity and directly depends on its gene expression [35, 37]. Considering that Ref-1 is a classic redox factor in preventing cells from being damaged by high concentrations of reactive oxygen species, we analyzed the correlation between BRAF mutation, APEX1 expression level, and NOX4 expression level in PTC, which represents higher levels of ROS to some extent. The results showed the expression level of NOX4 is positively correlated with the mutation state of BRAF and the expression level of APEX1. We speculated that in BRAF-mutated PTC, Ref-1 by BRAF mutations accompanied by high levels of ROS may be an important redox balance system caused to activate.

E3330, as a specific inhibitor of Ref-1 redox function, exerts an important antitumor effect on various tumors [38–40]. In this study, we proved that E3330 positively enhanced the treatment sensitivity and antitumor capacity of vemurafenib in PTC both in vitro and in vivo. We ultimately revealed another conceivable mechanism by which BRAF can maintain its activation status in the context of inhibitor treatment through the binding effect of the Ref-1 domain and the conformational change in Ref-1 caused by E3330 dissociating Ref-1 from the BRAF protein, which provides an opportunity for vemurafenib to enter the active pocket, thereby enhancing the inhibitory effect of vemurafenib; however, unfortunately, the definite mechanism is still not clear. Understanding the binding site and mode of action between Ref-1 and BRAF may provide a more useful therapeutic schedule. In addition, as we suppressed the expression of endogenous full-length Ref-1, we cannot exclude the possibility that the DNA repair activity of Ref-1 may also contribute to its sensitivity-promoting effect supporting vemurafenib treatment efficacy, and redox-targeting studies need to be performed in the future.

Currently, many mechanisms of vemurafenib resistance have been reported, among which the strong and acknowledged autophagy reaction caused by vemurafenib treatment clearly reduces the antitumor effect of this drug [23, 24, 41]. Autophagy can protect cancer cells by eliminating damaged organelles and recovering degradation products in normal cells, which may be the cause of resistance mediated via autophagy [42]. Numerous signaling pathways take part in the regulation of the autophagy process, such as the PI3K/AKT/mTOR pathway [43, 44], LKB1 (liver kinase B1)/AMPK/mTOR pathway [45–47], MAPK pathway [48, 49], p53 pathways (genotoxic stress) [50, 51], and so on. A close connection between vemurafenib and autophagy activated by the AMPK pathway has been reported many times. Sueda et al. [45] found that BRAF^V600E^ inhibitor treatment induced protective autophagy through AMPK activation, resulting in an attenuated drug effect on colorectal cancer cells. Niehr et al. [52] demonstrated that phosphorylated and activated AMPK after vemurafenib treatment could induce protective autophagy as our results showed.

Initially, in yeast, cells only used the autophagy program to fulfill autologous metabolic needs or renew certain organelles, thereby maintaining the vitality of cells suffering from nutritional deterioration [53]. However, in higher eukaryotes, autophagy plays much more complicated and multifunctional roles in regulating cell survival and death, especially in cancer cells [27, 29, 54, 55]. The commonly studied proteins closely related to the autophagy program mainly include LC3B and p62 [55–57], and their expression represents the differential autophagy status and output. The process of autophagosome fusion with acidic endosomes/lysosomes to form autolysates is defined as “autophagic flux” [54], which is one particular vital part of the process in the regulation of cell life. If contents are not cleared, scilicet eliminated or processed through autophagic flux, they accumulate and damage the cell, resulting in cellular senescence [29]. We observed in this study that combined inhibition with the two drugs studied appeared to be the phenomenon that enhanced AMPK activation, increased autophagy, which eventually led to the accumulation of cellular metabolic substrates or excessive consumption leading to senescence. Although senescence is often considered to be a temporary quiescent state to escape the immune system killing or to produce carcinogenic effects on surrounding cells through senescence-associated secretory phenotype (SASP), senescence induced by excessive autophagy and subsequent imbalance between energy consumption and supply caused by the autophagic turnover disorder of cellular constituents may lead to irreversible death of cells. But so far, the causal relationship between autophagy and senescence is still inconclusive. And in the combination group, we found that following the increase of phagocytosis and fusion, more and larger vacuoles were formed in the cells, occupying most of the cytoplasmic space, and there were few organelles left in the cells. These results indicate that autophagy and senescence are related events. The induction of senescence partly depends on the activation of autophagy, and excessive autophagy can lead to irreversible senescence events.

Several studies [25, 41, 44] have reported that dual inhibition of MAPK and autophagy caused by a MAPKi can specifically improve clinical efficacy in BRAF^V600E^ tumors; however, excessive autophagy can also cause cancer cells to undergo “autophagic cell death” or “type II programmed cell death” [42], and our research proved this point and provided another solution to vemurafenib resistance: BRAF inhibition and autophagy activation promoted by a MAPKi had a lethal effect on tumors. Given that the treatment inhibiting Ref-1 could further enhance the competitive blockade effect of vemurafenib on the constitutively active BRAF binding site, it is conceivable that the AMPK/mTOR pathway was further activated, thereby enhancing autophagy activation. Yuan et al. [58] combined AMPK activators and a BRAFi to perform experiments in melanoma, and they uncovered overlapping AMPK activation with superior antitumor effects for vemurafenib, which supports our conclusion to some extent.

## Conclusion

In this study, we found that the BRAF^V600E^ mutation upregulated the expression of Ref-1 in PTC patients, which might be one important reason for intrinsic resistance to BRAFi treatment. Combined treatment with vemurafenib and a Ref-1 redox inhibitor could enhance the antitumor capacity of vemurafenib by abolishing the maintenance effect on the BRAF protein and overloading autophagic flux both in vitro and in vivo; this approach is a potential strategy for BRAF-mutant PTC treatment.

## Materials and methods

### Clinical data and tissue samples

Formalin-fixed tissue samples from 178 PTC patients with complete clinicopathological information embedded in paraffin were made into 4 tissue microarrays, and 5-μm sections were cut. And 16 pairs of matched fresh tissues of PTC carcinoma and adjacent normal thyroid follicular tissue were collected in 2015 for total RNA and protein extraction. Informed consent was obtained from each patient to allow their information to be used. The research was performed with the approval of the Ethics Committee of Tianjin Medical University Cancer Institute and Hospital.

### Cell culture

The human TC cell lines K-1, BCPAP, and TPC-1 involved in this study all have STR identification certificates and are tested for mycoplasma contamination. BCPAP (BRAF^V600E^ mutation), K-1 (BRAF^V600E^ mutation), and TPC-1 (BRAF^wt^) human papillary thyroid carcinoma cells were purchased from Bestbay. BCPAP and TPC-1 cells were cultured in RPMI 1640 Medium (Gibco, c11875500bt) supplemented with 10% fetal bovine serum (Eallbio, u16001dc), 1% L-glutamine (Gibco, 25030081), 1% nonessential amino acid solution (Gibco, 11140050) and 1% penicillin-streptomycin (Gibco, 10378016, 100 U/mL penicillin, 100 μg/mL streptomycin). K-1 cells were cultured in Dulbecco’s Modified Eagle Medium (Gibco, c11995500bt) with the same supplementation used for BCPAP cells. The cells were maintained in an incubator set to 37 °C with 5% CO_2_.

### Drugs, reagents, and antibodies

Vemurafenib (a novel small molecular inhibitor against V600E, V600D, and V600R mutant cell lines, competing with ATP for the binding domain of BRAFV600E-mutated monomer) and E3330 (a quinone derivative, binding to a partially unfolded state of Ref-1 and increasing the formation of disulfide bonds between cysteine residues to convert Ref-1 into a folded state for inhibition of its redox function) were purchased from Selleck Chemical (Shanghai, China) and diluted in dimethyl sulfoxide (DMSO). An anti-GAPDH antibody was purchased from Genetex (GTX100118). Anti-Ref-1 (ab202894) and anti-Actin (ab8226) antibodies were purchased from Abcam. Anti-Caspase3 (#9664), anti-cleaved-Caspase3 (#9602), anti-PARP (#9532), anti-cleaved-PARP (#5625), anti-p62 (#23214), anti-LC3B (#3868), anti-E-cadherin (#14472), anti-Vimentin (#5741), anti-Bcl2 (#15071), anti-Bax (#5023), anti-Ki67 (#9449), anti-MEK (#4694), anti-p-MEK (#9127), anti-ERK (#4695), and anti-p-ERK (#4370) antibodies were purchased from Cell Signaling Technology. Anti-Survivin (AB3610) and anti-Flag (F1804) antibodies were purchased from Sigma.

### Immunohistochemistry

The expression of Ref-1 in papillary thyroid carcinoma was detected using conventional IHC. Lung metastatic tumor and liver sections from animals were stained with H&E for routine histological examination and morphometric analysis or IHC stained.

For H&E staining, briefly, tissue sections on coated slides were dewaxed with xylene and gradient alcohol after being incubated in an oven at 70 °C for 1 h, counterstained with H&E, dehydrated, and covered.

For IHC staining, briefly, tissue sections were dewaxed as indicated in the H&E staining procedure and then subjected to antigen retrieval by boiling in 10 mM sodium citrate (pH 6.0) at 130 °C for 3 min. The slides were then pretreated with a 3% solution of hydrogen peroxide for 30 min, rinsed, and incubated with 5% normal goat serum for 20 min as a blocking agent. The sections were incubated with a mouse anti-Ref-1 antibody (1:800; Abcam) at 4 °C overnight. On the next day, the slides were washed in PBS and incubated with a secondary antibody for 30 min at room temperature. All steps were preceded by rinsing the sections with PBS (pH 7.6). The chromogen was 3,3-diaminobenzidine (DAB). Hematoxylin was used for counterstaining, and dehydrated gum was used as the sealant; the slides were then observed, scored, and imaged. Staining intensity was scored by three professional pathologists in a double-blind way and criteria as follows: 0 (−), 1 (+), 2 (++), and 3 (+++). The degree of staining was categorized as 0 (0% staining), 1 (1–30% staining), 2 (31–60% staining), 3 (61–80% staining), and 4 (81–100% staining). The final score was determined by multiplying the staining intensity score and the staining degree score, ranging from 0 to 12. Samples with a final score of less than 3 were considered to have low expression, while those with a score of 4–12 were considered to have high expression.

### Quantitative real-time PCR

Total RNA was extracted from PTC cells and fresh tissue using TRIzol reagent (Life Technologies), and 2 μg of RNA was reverse transcribed into cDNA by using the High-Capacity cDNA Reverse Transcription Kit (Applied Biosystems). The cDNA was then used as a template for exponential amplification using SYBR Green/ROX qPCR Master Mix (Thermo Scientific). ACTB was used as an internal reference. The primers sequences of APEX1 and ACTB were as follows respectively: 5’-CAATACTGGTCAGCTCCTTCG-3’ and 5’-TGCCGTAAGAAACTTTGAGTGG-3’; 5’-GGAGAGATTGGCTTTCCTGGAC-3’ and 5’-CCTCATGCCAAATCCAAGGCTG-3’.

### Western blot analysis

Cells were cultured in 6-well plates at a density of 5 × 10^5^/mL per well and then treated with vemurafenib (10 μM) or/and E3330 (50 μM) for 24 h. The cells were washed with cold PBS three times and lysed in a RIPA solution (Solarbio LIFE SCIENCES) with the protease inhibitor phenylmethylsulfonyl fluoride (PMSF, Solarbio LIFE SCIENCES) (1 mM) for 30 min on ice. The supernatants were collected after cell lysates were centrifuged at 12,000 rad for 15 min at 4 °C. The protein concentrations were quantified using the BCA Protein Assay (Solarbio LIFE SCIENCES) according to the manufacturer’s instructions. Equal amounts (30 μg) of total protein were separated by sodium dodecyl sulfate–polyacrylamide gel electrophoresis (10–12%) and transferred to a 0.45-μm PVDF membrane. After blocking with 5% bovine serum albumin (BSA) in TBST buffer for 2 h at room temperature, the membranes were incubated with a primary antibody at 4 °C overnight. The membranes were washed three times with TBST buffer and then incubated with peroxidase (HRP)-conjugated secondary antibody for 1 h at room temperature. Specific antibody binding was detected with the Chemiluminescence Kit (Millipore, Plano, TX, USA). Fluorescent signals were detected by a luminescent image analyzer (C-Digit, Gene Company Limited, China).

### Cell viability and colony formation assays

A Cell Counting Kit-8 (CCK-8) assay (Dojindo, Kumamoto, Japan) was used according to the manufacturer’s instructions to measure the effects of drugs on the proliferation of papillary thyroid carcinoma cells. Briefly, BCPAP cells were seeded in 96-well cell plates (Corning Inc., Corning, USA) at a density of 1000 cells/well in 200 μL of culture medium and grown overnight. The next day, vemurafenib or/and E3330 were added to each well at an appropriate concentration, and cells were incubated for 0, 24, 48, 72, and 96 h. Then, 20 μL of CCK-8 reagent was added to each well and incubated for 3 h at 37 °C. Finally, the absorbance was measured with an enzyme-labeled instrument (Thermo) at 450/650 nm. The experiments were repeated at least three times.

For colony formation analysis, BCPAP and K-1 cells were seeded in 6-well cell plates (Corning Inc., Corning, USA) at a density of 500 and 1000 cells/well in 2 mL of culture medium, respectively, and then treated with vemurafenib or/and E3330 at an appropriate concentration for 24 h. In the drug treatment groups, the medium was replaced with a fresh medium after 14 days. Colonies were washed with phosphate-buffered saline (PBS) 3 times, fixed with 4% paraformaldehyde for 20 min, stained with 0.5% crystal violet for 15 min at room temperature, and then counted.

### Flow cytometric analysis

For analysis of apoptosis, BCPAP and K-1 cells were treated with 10 μM vemurafenib, 50 μM E3330, or their combination in 6-well plates for 24 h. Apoptosis rates were assessed by flow cytometry after staining with Annexin V/propidium iodide (BD Pharmingen, Franklin Lakes, USA). In brief, cells were harvested, washed three times with cold PBS, and resuspended in 100 μL of binding buffer at a density of 1 × 10^5^ cells/mL. Then, 5 μL of Annexin V and 5 μL of propidium iodide were added to the cells and incubated for 15 min in the dark at room temperature. Then, flow cytometry was performed, and the results were reported for three independent experiments. After administration of the above-indicated treatments, the cell cycle was also evaluated. In brief, cells were harvested, washed three times in cold PBS, fixed with 70% ethanol in PBS at 4 °C overnight, and then incubated with 100 μg/mL RNase A, 0.2% Triton X-100 and 50 μg/mL propidium iodide for 30 min at 4 °C in PBS. The cells were analyzed by flow cytometry (BD Biosciences FACSCanto II, USA). The results were reported for three independent experiments. For BrdU analysis, the FITC BrdU Flow Kit was used (BD Pharmingen, Franklin Lakes, USA). In brief, BCPAP and K-1 cells were treated as described above for 12 h, followed by careful addition of 10 µL of BrdU solution (1 mM BrdU in 1× PBS) directly to each mL of tissue culture medium and incubation of the treated cells for 12 h. The BrdU-pulsed cells were selected, and antibodies to specific cell surface markers were added in 50 µL of staining buffer. Then, the cells were washed and fixed before treatment with DNase to expose incorporated BrdU. Finally, BrdU and total DNA were stained with fluorescent antibodies and a 7-AAD solution for cell cycle analysis.

### Mitochondrial membrane potential assay

The JC-1 Assay Kit (Beyotime, Beijing, China) was used to measure alterations in the mitochondrial membrane potential according to the manufacturer’s instructions. Cells were seeded in six-well plates at a density of 5 × 10^5^/mL and then treated with vemurafenib or/and E3330 at concentrations of 10 μM or/and 50 μM for 24 h. Then, 100 μL of JC-1 staining solution was added to 1 mL of culture medium and incubated for 20 min at 37 °C in a CO_2_ incubator. The samples were analyzed by flow cytometry, with JC-1 aggregates measured in the FL-2 channel and green fluorescence (JC-1 monomers) measured in the FL-1 channel (BD Biosciences).

### Electron microscopy (EM)

EM was carried out as previously described^72^. Briefly, DMSO- and drug-treated cells were fixed overnight at 4 °C in 2.5% glutaraldehyde, before being post-fixed with 1% OsO_4_ for 1 h. Cells were then dehydrated in a graded ethanol series and embedded in Agar 100 epoxy resin. After finished slicing, stained first with uranyl acetate followed by lead citrate. Sections were observed and photographed under a Philips CM10 Transmission Electron Microscope.

### Immunofluorescence

Cell slides were prepared before the experiment. After 8 h, the slides containing cells that had migrated were immersed in PBS three times for 3 min each time. The scaffolds were fixed with 4% paraformaldehyde for 20 min, permeabilized with 0.5% Triton X-100 at room temperature for 15 min, and sealed with 5% BSA for 1 h at room temperature. The blocking solution was absorbed by the absorbent paper, and a sufficient amount of diluted primary antibody was added to each slide and incubated in a humidified box overnight at 4 °C. The next day, the slides were washed 3 times with TBST, and a diluted fluorescent secondary antibody was added, followed by incubation at room temperature for 1 h in the dark. The slides were washed 3 times using TBST and sealed using 5 μL of antifade mounting medium with DAPI (Invitrogen). Images were acquired under a fluorescence microscope.

### Cell senescence staining experiment

A cellular senescence β-galactosidase staining kit (Beyotime, Beijing, China) was used to measure cellular aging according to the manufacturer’s instructions. Cells were plated in 6-well plates and treated with vemurafenib or/and E3330 or/and HCQ at an appropriate concentration for 24 h. Then, 1 mL of β-galactosidase staining fixative was added, and the mixture was fixed at room temperature for 15 min. The cells were washed with PBS, and 1 mL of staining solution was added per well. The 6-well plate was sealed with plastic wrap to prevent evaporation and then incubated overnight at 37 °C. The plate was observed under an ordinary light microscope, and images were acquired.

### Studies in vivo

The experimental protocol on nude mice was approved by the Ethics Committee of Tianjin Medical University Cancer Institute and Hospital. All procedures involving animals and their care were conducted in conformity with institutional guidelines in compliance with national and international laws and policies. Female BALB/c nude mice (5 weeks old) were purchased from the Tianjin Institute of Health and Environmental Medicine. Mice were randomly divided into control groups and different experimental groups by the random number method. The BCPAP cell line was suspended in PBS and injected into mice (seven mice per group) subcutaneously at a concentration of 1 × 10^6^ cells/mouse to establish a xenograft model and the BCPAP cell line subjected to luciferase labeling were suspended in PBS and injected into mice (five mice per group) via the tail vein at a concentration of 1 × 10^6^ cells/mouse to establish lung metastasis animal model. After 2 weeks, the mice were fed 2% hydroxymethyl cellulose (HMC), 20 mg/kg vemurafenib in 1% HMC or/and 50 mg/kg E3330 in 1% HMC once a day for 3 weeks. Subcutaneous tumor and metastatic tumor size were respectively measured every 3 days using vernier caliper and live animal imaging system, and body weight was measured every 3 days. At the final time point (3 weeks), the mice were sacrificed, and the tumors, lungs, livers, and kidneys were removed and fixed with 4% paraformaldehyde.

### Statistical analysis

Statistical analysis was performed using the SPSS version 17.0 software and GraphPad Prism 7.0 software. For measurement data, the values are expressed as the mean ± SEM of at least three separate experiments. Categorical count data are shown as the number of cases. Student’s *t* test (two-sided) and *χ*^2^ tests were used to calculate the significance of differences between groups. Kaplan–Meier survival analysis was performed for the Ref-1 high- and low-risk groups to evaluate the recurrence of patients. Statistical significance is indicated by *P* < 0.05.

## Supplementary information

Checklist

Supplemental figures

## Data Availability

The data sets used and/or analyzed during the current study are available from the corresponding author on reasonable request.
