# Supplementary material for: Pharmacological inhibition of Ref-1 enhances the therapeutic sensitivity of papillary thyroid carcinoma to vemurafenib
Source: Cell Death Dis. 2022 Feb 8;13(2):124. doi: 10.1038/s41419-022-04550-0 (PMC8825860; doi:10.1038/s41419-022-04550-0)
Supplement: Supplementary file 2 — Supplemental figures [file 41419_2022_4550_MOESM2_ESM.docx]

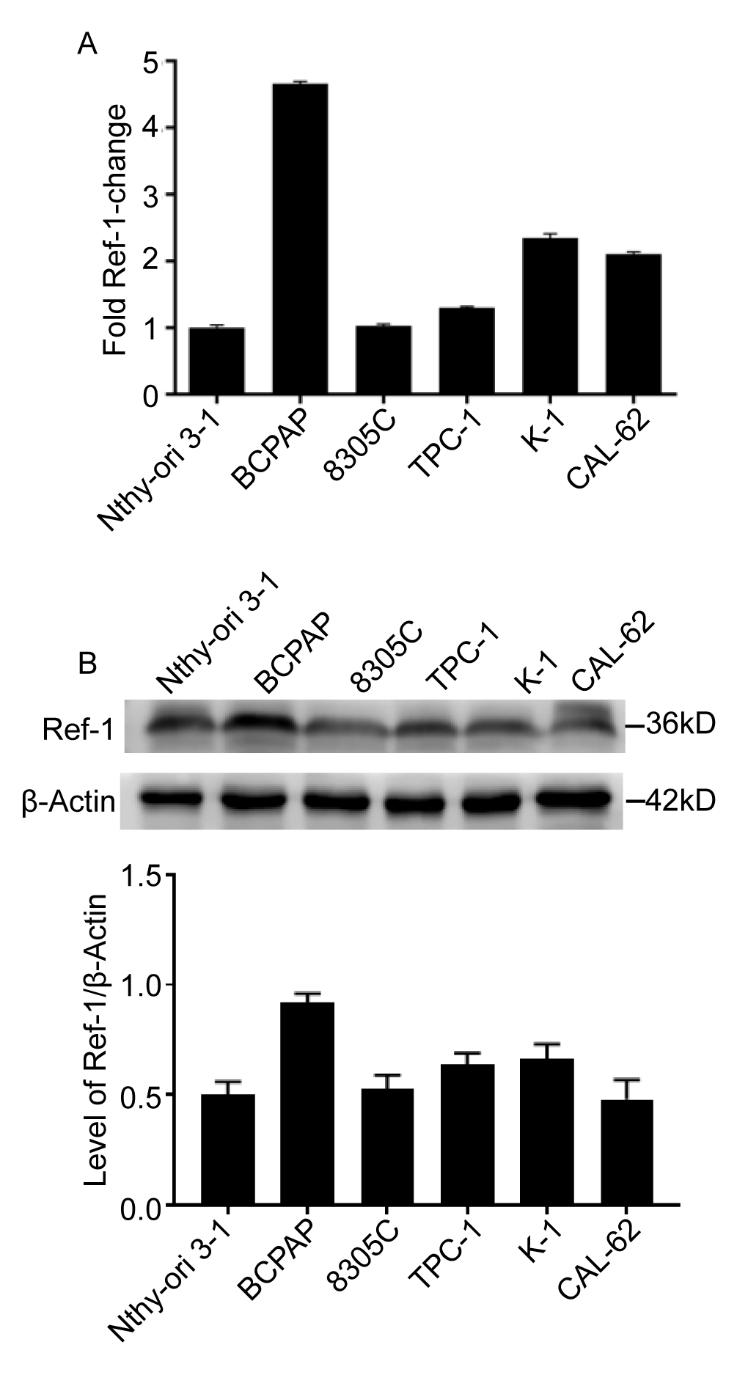


**Supplemental figure 1: Ref-1 expression was upregulated in PTC cell lines harboring BRAF^V600E^.**

A, B. Ref-1 expression detection in normal thyroid and thyroid cancer cell lines by qPCR and Western blotting.


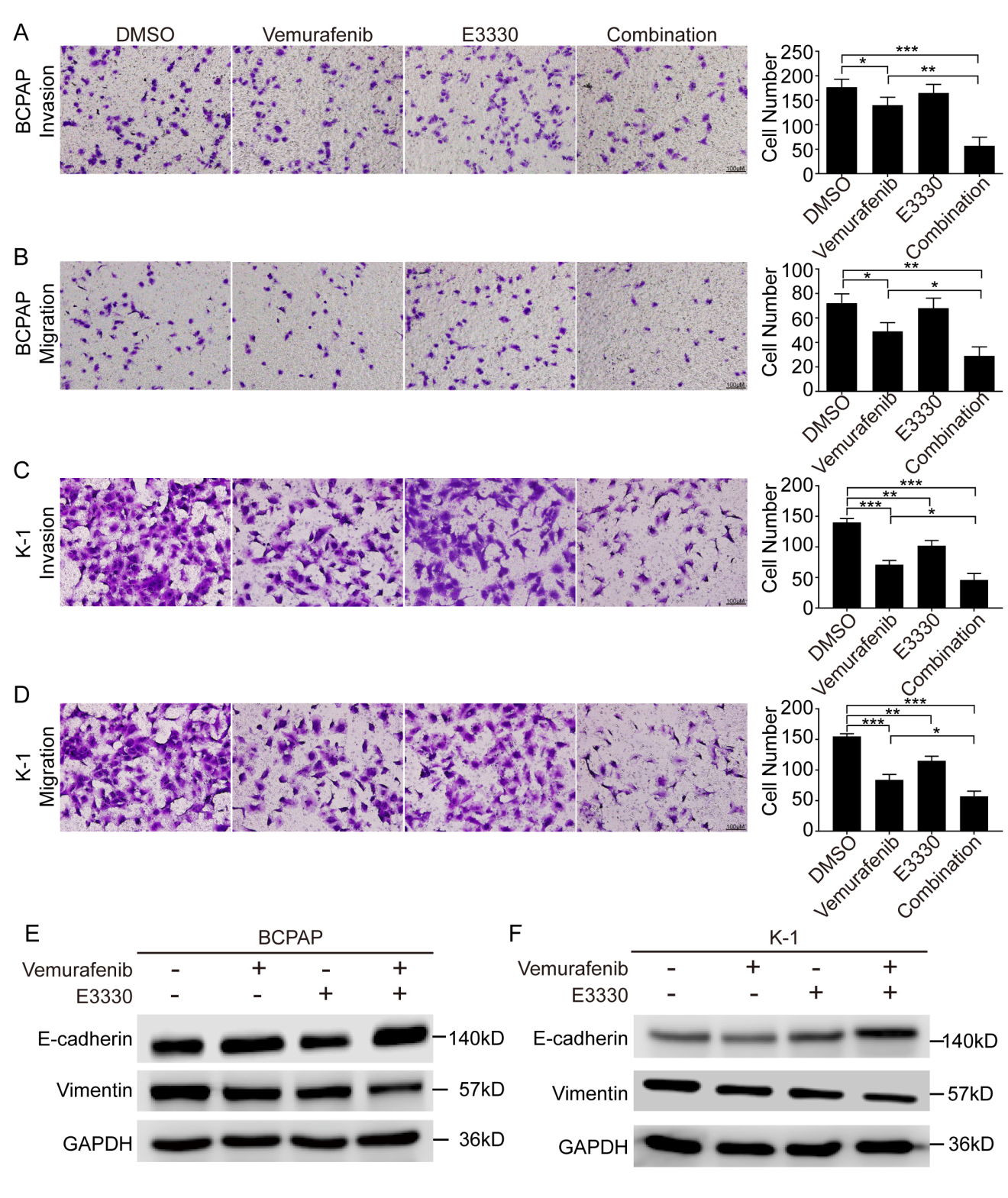


**Supplemental figure 2: E3330 enhanced the inhibition of invasion and migration by vemurafenib in BCPAP and K-1 cells.**

A, B. Cell invasion and migration ability detection by transwell assays in BCPAP cell line. C, D. Cell invasion and migration ability detection by transwell assays in K-1 cell line. E,F. Epithelial-mesenchymal transition associated protein expression detection by western blotting in BCPAP and K-1 cell lines. BCPAP and K-1 cell lines were pretreated with DMSO, vemurafenib (10μM), E3330 (50μM), and combination (10μM vemurafenib+50μM E3330) respectively. **P* < 0.05, ***P* < 0.01, ****P* < 0.001.


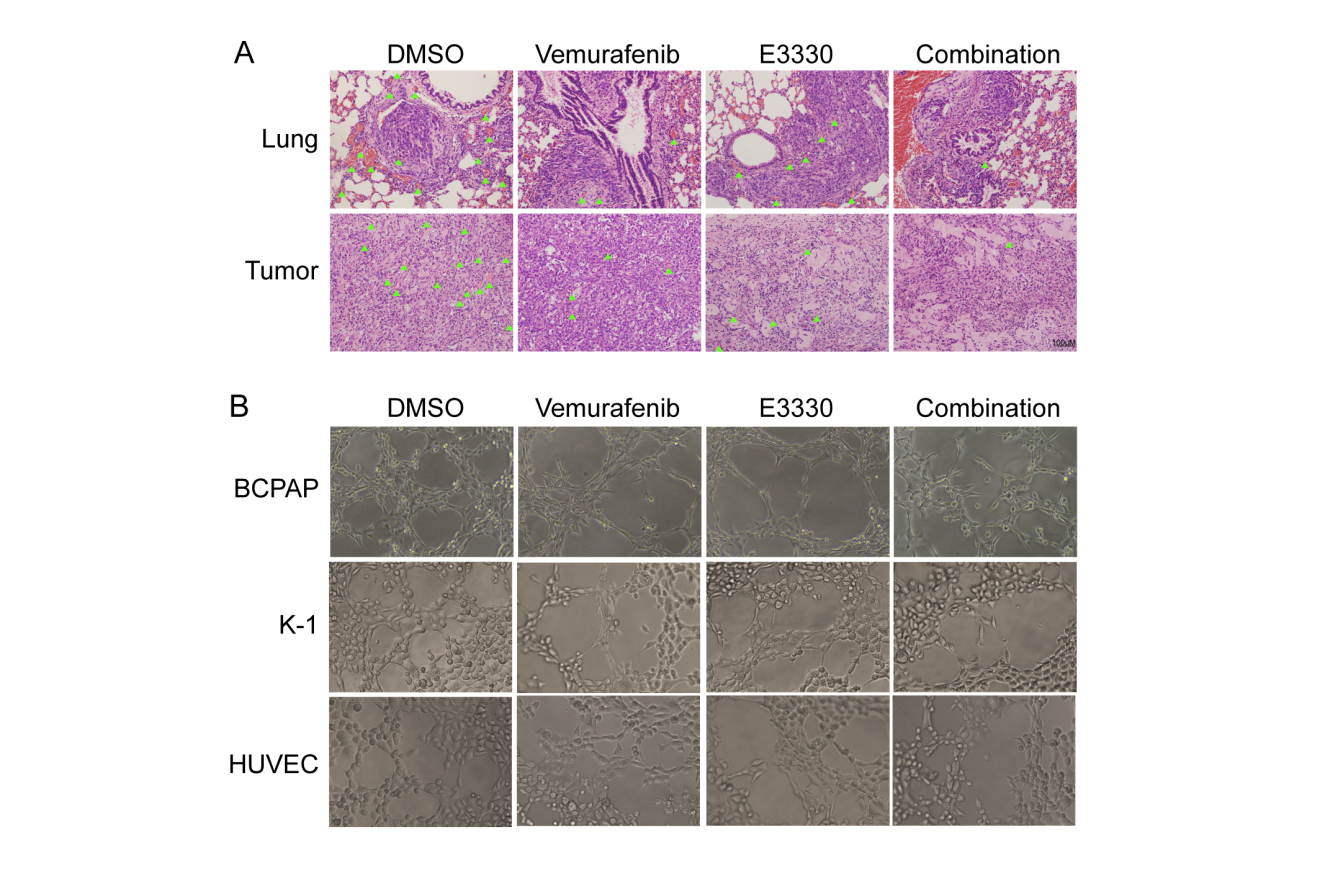


**Supplemental figure 3:** **E3330 enhanced the inhibition of angiogenesis by vemurafenib in BCPAP and K-1 cell lines.**

1. Representative H&E staining of dissected mouse subcutaneous tumors after HMC, vemurafenib, E3330, or combination (vemurafenib+E3330) treatment at and 21 days. The green triangles indicate blood vessel. B. Angiogenesis ability detection by angiogenesis assay in BCPAP, K-1 and HUVEC cell lines treated with DMSO, vemurafenib (10μM), E3330 (50μM), and combination (10μM vemurafenib+50μM E3330) respectively.


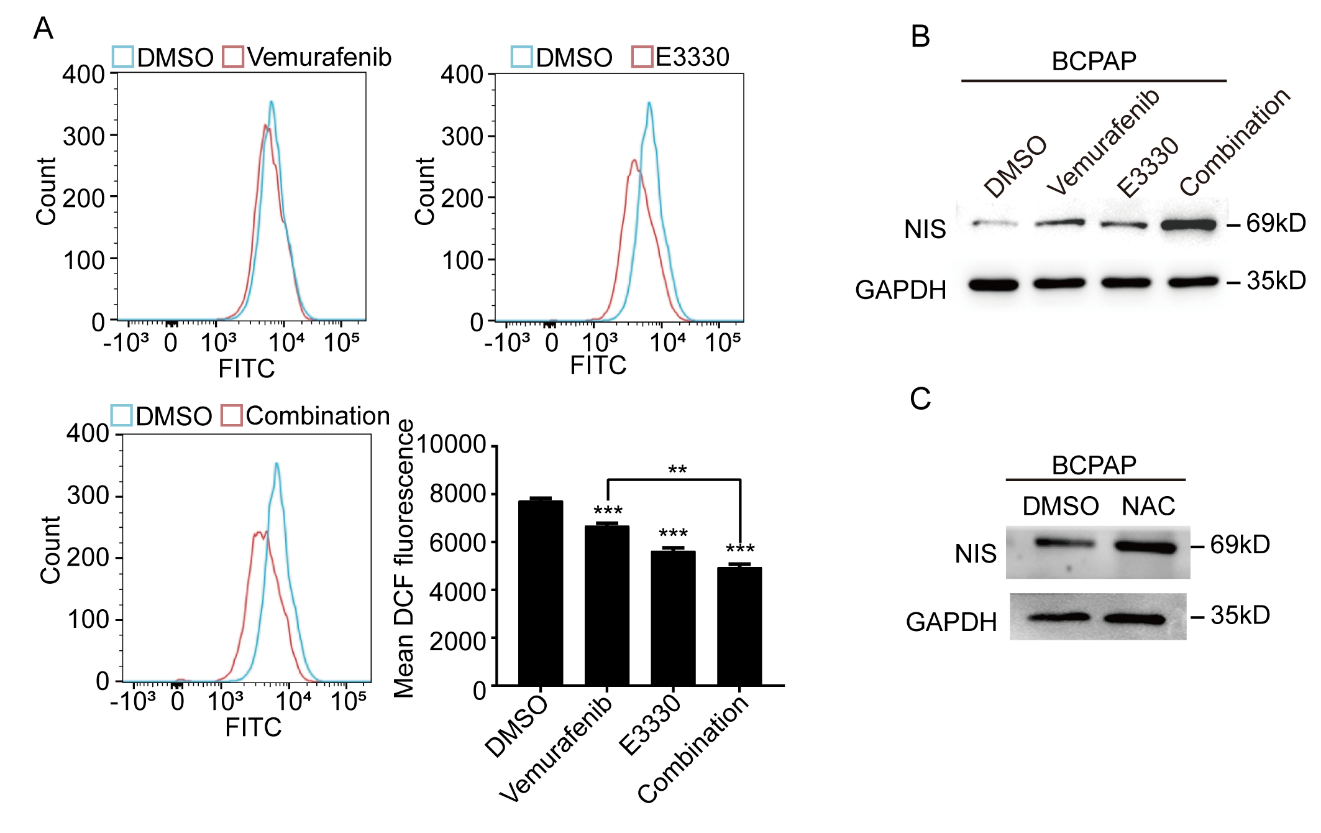


**Supplemental figure 4:** **E3330 enhanced the differentiation promoting effect by vemurafenib in BCPAP cell line.**

A. ROS generation detection by DCFH-DA probe in BCPAP cell line treated with DMSO, vemurafenib (10μM), E3330 (50μM), and combination (10μM vemurafenib+50μM E3330) respectively. B. NIS expression detection by western blot in BCPAP cell line treat with treated with DMSO, vemurafenib (10μM), E3330 (50μM), and combination (10μM vemurafenib+50μM E3330) respectively. C. NIS expression detection by western blot in BCPAP cell line treat with treated with DMSO and NAC (5μM).

NAC: N-Acetyl-L-cysteine, ***P* < 0.01, ****P* < 0.001.

**Supplemental Materials and Methods**

**Transwell of migration and invasion assay**

Cell migration and invasion assays were performed with polycarbonate membrane filter inserts with 8 μm pores (Merck Millipore, USA) in 24-well chamber plates. The transwell chambers were precoated with (invasion assay) or without (migration assay) one Matrigel diluted in eight 1640 (BD Biosciences, USA). In brief, the bottom chamber was filled with 500 μL of culture medium containing 10% FBS, and the upper chamber was filled with 200 μL of 20,000 cell culture medium without FBS. The cells were incubated for 16 h (BCPAP) and 18 h (K-1) with 5% CO_2_ at 37 °C in migration assay and were incubated for 48 h in invasion assay. Then, cells at the bottom surface of the membrane were fixed in 4% polyoxymethylene for 15 min and stained with 0.05% crystal violet for 30 min. The mean numbers of three preselected microscopic fields were counted.

**Angiogenesis assay**

We simulated angiogenesis using BCPAP, K-1, and HUVEC cell lines to evaluate the effect of drug treatment on the ability of tumor cells or endothelial cells to form capillary-like structures (tubes). Cells were pretreated with DMSO/Vemurafenib/E3330/ Combination for 24 h. Matrigel was evenly added to a 96-well plate with 50μL/well and then placed in cell incubator at 37 °C. After solidification of matrigel, 2 × 10^4^ cells were plated carefully each well, and then observed under microscope every 1 h until obvious blood vessel formation could be seen.

**Reactive oxygen species (ROS) detection**

Intracellular ROS production was detected by using peroxide sensitive fluorescent probe DCFH-DA in the ROS Detect Assay Kit (Beyotime, Beijing, China). The cells were incubated in 6-well plates and treated with 10 μM vemurafenib and/or 50 μM E3330. Then the cells were incubated with DCFH-DA at 20 μM in fetal bovine serum 1640 / DMEM at 37℃ for 30 min. The cells were washed with pre-cooled 1640 / DMEM for 3 times and collected for the detection of ROS level by flow cytometry.
